# Supplementary figures and images for: Fixation can change the appearance of phase separation in living cells
Source: eLife. 2022 Nov 29;11:e79903. doi: 10.7554/eLife.79903 (PMC9817179; doi:10.7554/eLife.79903)

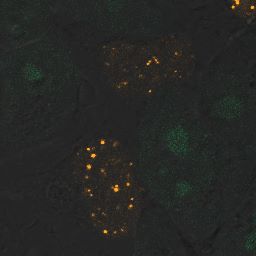

Supplement: Source code 1. [file elife-79903-code1.zip › Processing Scripts/thumbnail.jpg]
