# Supplementary material for: Fixation can change the appearance of phase separation in living cells
Source: eLife. 2022 Nov 29;11:e79903. doi: 10.7554/eLife.79903 (PMC9817179; doi:10.7554/eLife.79903)

COPASI

0.1 Programmatically Accessing COPASI

Shawn Irgen-Gioro 12/27/2021

We will start out with exploring the behavior of this 4 state kinetic model:

$$\text{Bound} \xrightarrow{k_1} \text{Unbound} \tag{1}$$

$$\text{Bound} \xleftarrow{k_2} \text{Unbound} \tag{2}$$

$$\text{Bound} \xrightarrow{k_3} \text{Fixed Bound} \tag{3}$$

$$\text{Bound} \xrightarrow{k_4} \text{Fixed Unbound} \tag{4}$$

where bound and unbound are in a reversable dynamic equilibrium. Before $t = 0$, bound and unbound are in steady state equilibrium. $t = 0$ represents the time that a fixative is added. Since k3 and k4 are irrevesable reactions, we expect that as $t = \infty$, all the final population is in fixed bound or fixed unbound. Thus, the main read out will be the comparison of the amount of fixed bound at $t = \infty$ to bound at $t = 0$.

I'm calling proteins that are in a hub "bound" and proteins that are not in a hub "unbound."

Start by importing packages. basico is a wrapper for COPASI

[1]:
```
from basico import *
import matplotlib
import matplotlib.pyplot as plt
import seaborn as sns
import numpy as np
```

I previously have used COPASI to run some more basic numeric simulations. We will use this as a starting point

[2]:
```
load_model('Z:\\Data\\20210916\\Kinetic Simulations.cps')
```

[2]:
```
<CDataModel "Root">
```

The previoulsy setup reaction parameters and species are loaded here as pandas dataframes for ease of access. The parameters loaded are shown below:

[3]:

```
reactions = get_reaction_parameters()
species = get_species()
display(reactions)
display(species)
```

```
                                 value                     reaction   type  \
name
(Bound = Unbound).k1              0.10             Bound = Unbound  local
(Bound = Unbound).k2              0.03             Bound = Unbound  local
(Bound -> Fixed).k1               0.50              Bound -> Fixed  local
(Unbound -> "Fixed Unbound").k1   2.50  Unbound -> "Fixed Unbound"  local

                                mapped_to
name
(Bound = Unbound).k1
(Bound = Unbound).k2
(Bound -> Fixed).k1
(Unbound -> "Fixed Unbound").k1
```

```
               compartment       type   unit  initial_concentration  \
name
Bound          compartment  reactions  mol/l                   0.23
Unbound        compartment  reactions  mol/l                   0.77
Fixed Bound    compartment  reactions  mol/l                   0.00
Fixed Unbound  compartment  reactions  mol/l                   0.00

               initial_particle_number initial_expression expression  \
name
Bound                     1.385092e+23
Unbound                   4.637048e+23
Fixed Bound               0.000000e+00
Fixed Unbound             0.000000e+00

               concentration  particle_number  rate  particle_number_rate  \
name
Bound                    NaN              NaN   0.0                   0.0
Unbound                  NaN              NaN   0.0                   0.0
Fixed Bound              NaN              NaN   0.0                   0.0
Fixed Unbound            NaN              NaN   0.0                   0.0

                        key sbml_id
name
Bound          Metabolite_0
Unbound        Metabolite_1
Fixed Bound    Metabolite_2
Fixed Unbound  Metabolite_3
```

We will now set some of the parameters. The initial concentrations will be determined from the k_1 and k_2, (remembering that the ratio of concentrations at equilibrium $[Unbound]_{eq}/[Bound]_{eq} =$

$K_{eq} = k_1/k_2$). We will always normalize the total concentration to be 1.

The preivous names of the rates are not consistent with the naming here. I will stick with the k1/k2/k3/k4 naming for the script, and only use the previous naming for inputting the rates into the model

[4]:
```
#set rates
k1 = 10
k2 = 5
k3 = .5
k4 = 2.5

#Set steadystate populations
B0 = 1 #Bound Initial. Will normalize in a second. Using 1 just to make the␣
↪next step easy
U0 = k1/k2
B0 = B0/(U0+1)
U0 = U0/(U0+1)
ratio_0 = U0/B0
```

Input these parameters in the model

[5]:
```
set_reaction_parameters('(Bound = Unbound).k1', value=k1)
set_reaction_parameters('(Bound = Unbound).k2', value=k2)
set_reaction_parameters('(Bound -> Fixed).k1', value=k3)
set_reaction_parameters('(Unbound -> "Fixed Unbound").k1', value=k4)
```

[6]:
```
set_species('Bound', initial_concentration=B0)
set_species('Unbound', initial_concentration=U0)
set_species('Bound', initial_concentration=B0)

#get_species().initial_concentration
```

Run the simulation, the plot really is just to check that we're really at equilibrium. Might need to make this longer if we play with our k3/k4 a lot

[7]:
```
result = run_time_course(duration=10)
result.plot();

#we just want the ratio of the last time point
FB_inf = result.iloc[-1]['Fixed Bound']
FU_inf = result.iloc[-1]['Fixed Unbound']
ratio_inf = FU_inf/FB_inf
delta_ratio = (ratio_inf - ratio_0)/ratio_0;
```

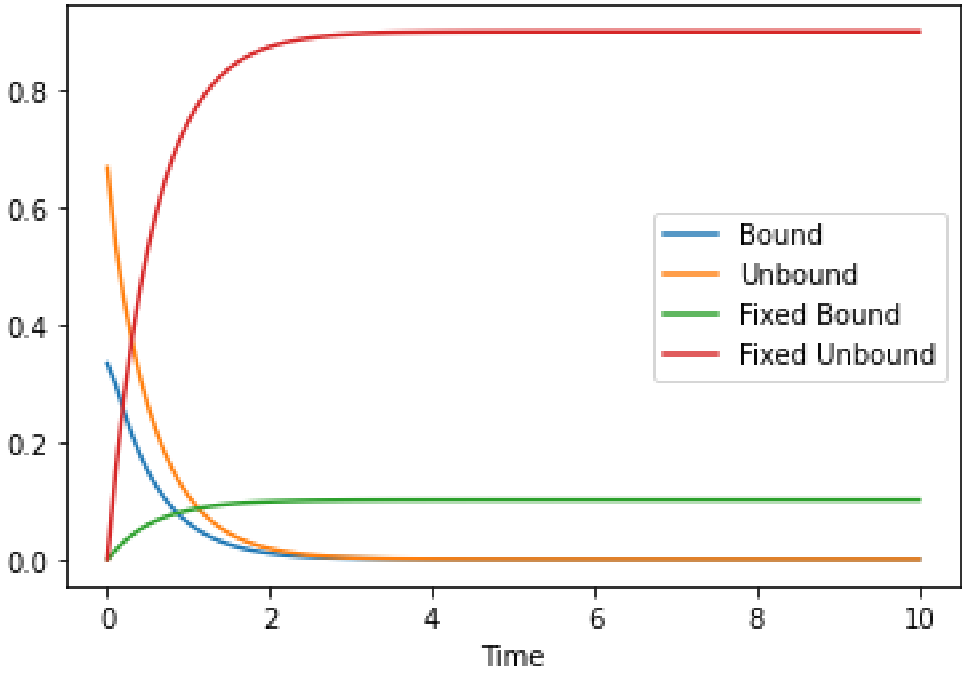

Going to put this all into a for loop to look for dependence of initial concentration

[8]:
```
#we're going to keep k1 and k2 in a similar numeric range
min_kval = .1;
max_kval = 10;
numpoints = 50;
k_range= np.sort(np.linspace(min_kval,max_kval,num=numpoints));

delta_bound = np.zeros(len(k_range))
B_int = np.zeros(len(k_range))

for ii in range(len(k_range)):
    k1 = k_range[ii]; #print('k1 = ' + str(k1));
    k2 = k_range.max()-k1+.001; #print('k2 = ' + str(k2));
    k3 = 2
    k4 = 1

    B0 = 1 #Bound Initial. Will normalize in a second. Using 1 just to make the␣
 ↪next step easy
    U0 = k1/k2
    B0 = B0/(U0+1); #print('B0 = ' + str(B0));
    U0 = U0/(U0+1); #print('U0 = ' + str(U0));
    ratio_0 = U0/B0
#     print('ratio_0 = '+ str(ratio_0)) #to check if the noramlization is␣
 ↪working
```

```
    set_reaction_parameters('(Bound = Unbound).k1', value=k1)
    set_reaction_parameters('(Bound = Unbound).k2', value=k2)
    set_reaction_parameters('(Bound -> Fixed).k1', value=k3)
    set_reaction_parameters('(Unbound -> "Fixed Unbound").k1', value=k4)

    set_species('Unbound', initial_concentration=U0)
    set_species('Bound', initial_concentration=B0)
    set_species('Unbound', initial_concentration=U0) #i dont know what the␣
 ↪issue is, but i have to run it twice to get it to work

    result = run_time_course(duration=500)
#     result.plot();

    #we just want the ratio of the last time point
    FB_inf = result.iloc[-1]['Fixed Bound']
    FU_inf = result.iloc[-1]['Fixed Unbound']
    ratio_inf = FU_inf/FB_inf
#     print('ratio_inf =' + str(ratio_inf))
#     print('next')
    B_int[ii] = B0;
    delta_bound[ii] = FB_inf-B0 #looking specifically at the change of the␣
 ↪concentration of In Hub

#print(delta_bound)
plt.plot(B_int,delta_bound)
plt.ylabel('Delta Change In Hub')
plt.xlabel('Starting Hub Fraction')
plt.savefig("Starting Hub Fraction.svg")
```

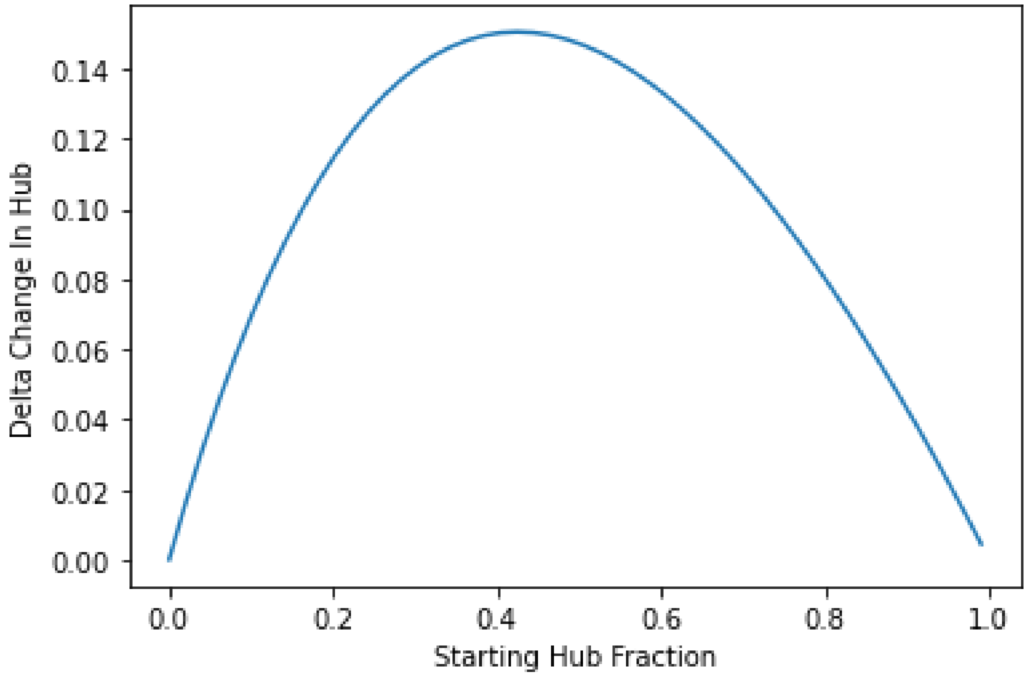

Here, we show that as a function of intial "in hub" concentration, fixation is most effective for intermediate bound fractions

0.2 Looping over different K1=K2 dynamics

0.2.1 In a sense, what this is looking at is the effect of the rate of PFA fixation

we're going to add that entire loop into another loop to look for dependence of ratio of k1/k2 relative to k3 and k4.

Since differnet processes occur in the cell at different timescales, the relative rate of fixation and intrisic dynamics is investigated here.

Additionally, by keeping the ratio of K1/K2 constant and changing the rate relative to the fixation rates, we can continue plotting as a function of starting hub fraction

[9]:
```
#define range of k1/k2 that have constant ratios, but larger or slower compared␣
↪to k3/k4
#since we're going to be plotting as a function of starting in-hub, then we␣
↪might as well just keep that constant so we don't have to interpolate after

numpoints = 50;

BoundRatios = np.linspace(1/10,99.9,numpoints)/100 #ratio of 1/10, 100/1, 100␣
↪points in between. we're defining this as U/B still
```

```
numkmax = 60;
startkval = 0.3;
endkval = 120;
kmaxs = np.logspace(np.log10(startkval),np.log10(endkval),numkmax)

delta_bound = np.zeros([numkmax,numpoints])

for jj in range(numkmax):
    kmax = kmaxs[jj]
    #print(jj)
    for ii in range(numpoints):

        B0 = BoundRatios[ii] #we start by defining the equilibrium states, and␣
 ↪work backwards
        U0 = 1-B0;
    #     print('B0 = ' + str(B0));
    #     print('U0 = ' + str(U0));


        k1 = U0*kmax; #print('k1 = ' + str(k1));
        k2 = B0*kmax; #print('k2 = ' + str(k2));
        k3 = 5
        k4 = 1

        set_reaction_parameters('(Bound = Unbound).k1', value=k1)
        set_reaction_parameters('(Bound = Unbound).k2', value=k2)
        set_reaction_parameters('(Bound -> Fixed).k1', value=k3)
        set_reaction_parameters('(Unbound -> "Fixed Unbound").k1', value=k4)

        set_species('Unbound', initial_concentration=U0)
        set_species('Bound', initial_concentration=B0)
        set_species('Unbound', initial_concentration=U0) #i dont know what the␣
 ↪issue is, but i have to run it twice to get it to work

        result = run_time_course(duration=500)
#          result.plot();

        #we just want the ratio of the last time point
        FB_inf = result.iloc[-1]['Fixed Bound']
        FU_inf = result.iloc[-1]['Fixed Unbound']
        ratio_inf = FU_inf/FB_inf
    #     print('ratio_inf =' + str(ratio_inf))
    #     print('next')
        delta_bound[jj,ii] = FB_inf-B0 #looking specifically at the change of␣
 ↪the concentration of In Hub

    # print(delta_bound)
```

[10]:

```python
fig, ax = plt.subplots()
im = ax.imshow(delta_bound,cmap=plt.get_cmap('Blues'))
# im = sns.heatmap(data=delta_bound)

# Show all ticks and label them with the respective list entries
ax.set_xticks(range(len(BoundRatios)),labels=np.round(BoundRatios,2))
ax.set_yticks(range(len(kmaxs)),labels=np.round(kmaxs/(k3+k4),2))
plt.xticks(rotation = 45)
plt.colorbar(im)
ax.set_ylabel('mean(k1/k2)/mean(k3/k4)')
ax.set_xlabel('Starting Bound Fraction')

fig.set_size_inches(18.5, 10.5)
plt.savefig("Fixation Speed.svg")
```

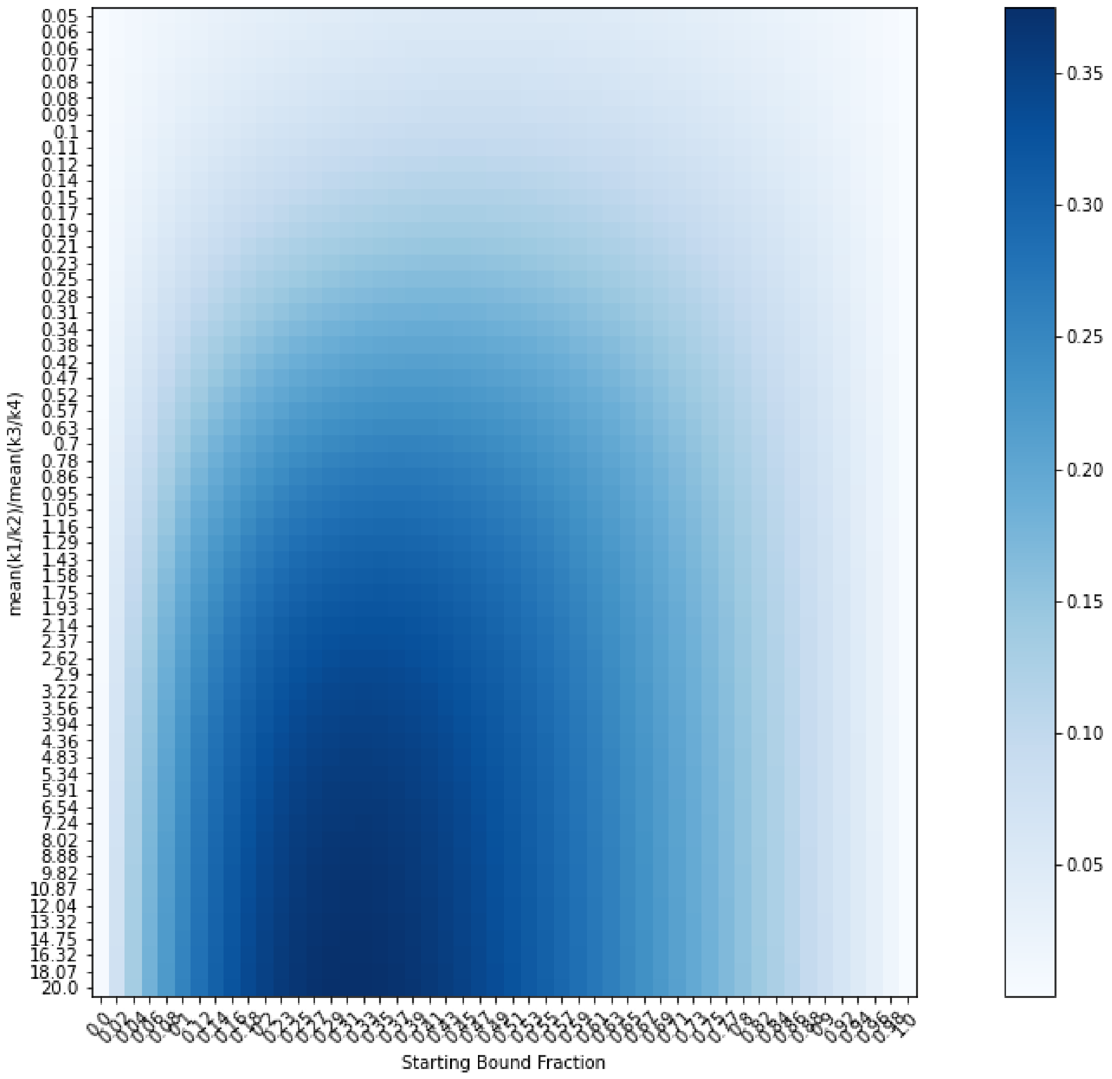

When the starting bound fraction is 0, that means that the k1 is very large, pushing all the population to unbound. This means that when fixation occurs, almost no bound exists to get fixed. Similarly at high concentrations, it is hard to increase the bound fraction any more than already exists. The effect of fixation speed is seen as faster fixation decreases the chage in bound fraction.

0.3 Looping over k3/k4 ratios

The thought is that the fixation rates are not constant for different types of proteins. For example, as written in DOI: 10.1074/jbc.R115.651679, “N-terminal amino groups may be less available and side chains are less accessible to formaldehyde crosslinking due to protein tertiary structure in native proteins.” Well defined proteins have fixed tertiary structures, creating steric hinderance so that IDR proteins without tertiary structure have more of their amino acids avaiable to be cross linked.

[11]:
```
#we're going to keep k1 and k2 in a similar numeric range
#to do, this is defined as a set of numbers. need to define this like a ratio␣
↪like k3 and k4 and keep constant total value
min_kval = .01;
max_kval = 1;
numpoints = 50; #number of points along k1/k2
k_range= np.sort(np.linspace(min_kval,max_kval,num=numpoints));

maxfixratio = 5;
fix_num = 50; #number of points along k3/4
fixnumerator = np.linspace(1,maxfixratio,fix_num)
fixdenominator = np.linspace(maxfixratio,1,fix_num)
fix_k_range = (fixnumerator/fixdenominator)

delta_bound = np.zeros([fix_num,numpoints])
B_int = np.zeros(numpoints)
fix_ratio = np.zeros(fix_num)

for jj in range(fix_num):
    for ii in range(numpoints):

        k1 = k_range[ii]; #print('k1 = ' + str(k1));
        k2 = k_range.max()-k1+.001; #print('k2 = ' + str(k2));

        k3 = fix_k_range[jj]
        k4 = (1./fix_k_range[jj])
        k3 = (k3/(k3+k4))* maxfixratio
        k4 = (k4/(fix_k_range[jj]+k4))* maxfixratio #this is so that the total␣
↪k3+k4 stays constant
#         print('k3 = ' + str(k3));
#         print('k4 = ' + str(k4));
```

```
        B0 = 1 #Bound Initial. Will normalize in a second. Using 1 just to make␣
↪the next step easy
        U0 = k1/k2
        B0 = B0/(U0+1); #print('B0 = ' + str(B0));
        U0 = U0/(U0+1); #print('U0 = ' + str(U0));
        ratio_0 = U0/B0
    #     print('ratio_0 = '+ str(ratio_0)) #to check if the noramlization is␣
↪working

        set_reaction_parameters('(Bound = Unbound).k1', value=k1)
        set_reaction_parameters('(Bound = Unbound).k2', value=k2)
        set_reaction_parameters('(Bound -> Fixed).k1', value=k3)
        set_reaction_parameters('(Unbound -> "Fixed Unbound").k1', value=k4)

        set_species('Unbound', initial_concentration=U0)
        set_species('Bound', initial_concentration=B0)
        set_species('Unbound', initial_concentration=U0) #i dont know what the␣
↪issue is, but i have to run it twice to get it to work

        result = run_time_course(duration=500)
#         result.plot();

        #we just want the ratio of the last time point
        FB_inf = result.iloc[-1]['Fixed Bound']
        FU_inf = result.iloc[-1]['Fixed Unbound']
        ratio_inf = FU_inf/FB_inf
    #     print('ratio_inf =' + str(ratio_inf))
    #     print('next')
        B_int[ii] = B0;
        delta_bound[jj,ii] = FB_inf-B0 #looking specifically at the change of␣
↪the concentration of In Hub
    fix_ratio[jj] = k3/k4
    #print(jj)
```

[12]:

```
fig, ax = plt.subplots()
im = ax.imshow(delta_bound,cmap=plt.get_cmap('RdBu'))
# im = sns.heatmap(data=delta_bound)

# Show all ticks and label them with the respective list entries
ax.set_xticks(range(len(B_int)),labels=np.round(B_int,2))
ax.set_yticks(range(len(fix_ratio)),labels=np.round(fix_ratio,2))
plt.xticks(rotation = 45)
plt.colorbar(im)
ax.set_ylabel('k3/k4')
ax.set_xlabel('Starting Bound Fraction')
```

```
fig.set_size_inches(18.5, 10.5)
plt.savefig("Relative Fixation Rate.svg")
```

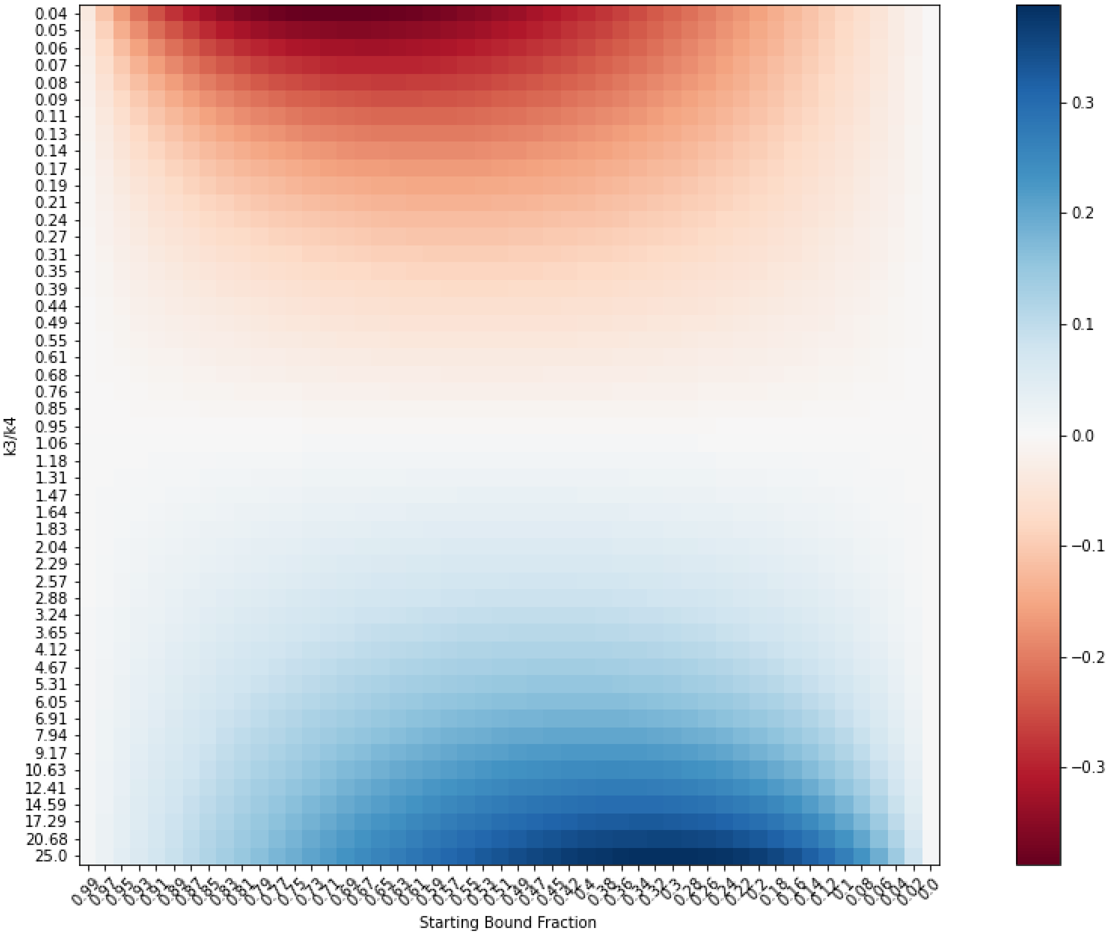

Supplement: Source code 2. [file elife-79903-code2.zip › Kinetic Simulation/COPASI_SI.pdf]
